# High concurrent validity between digital and analogue algometers to measure pressure pain thresholds in healthy participants and people with migraine: a cross-sectional study

**DOI:** 10.1186/s10194-021-01278-8

**Published:** 2021-07-12

**Authors:** René F. Castien, Michel W. Coppieters, Tom S. C. Durge, Gwendolyne G. M. Scholten-Peeters

**Affiliations:** 1 Faculty of Behavioural and Movement Sciences, Vrije Universiteit Amsterdam, Amsterdam Movement Sciences, Van der Boechorststraat 9, 1081 BT Amsterdam, The Netherlands; 2 Amsterdam Public Health Research Institute, Amsterdam University Medical Center, Amsterdam, The Netherlands; 3 Health Care Centre Haarlemmermeer, Hoofddorp, The Netherlands; 4 Menzies Health Institute Queensland, Griffith University, Brisbane & Gold Coast, Australia

**Keywords:** Mechanical hyperalgesia, Headache, Quantitative sensory testing, Rehabilitation, Musculoskeletal health

## Abstract

**Background:**

Pressure pain thresholds (PPTs) are commonly assessed to quantify mechanical sensitivity in various conditions, including migraine. Digital and analogue algometers are used, but the concurrent validity between these algometers is unknown. Therefore, we assessed the concurrent validity between a digital and analogue algometer to determine PPTs in healthy participants and people with migraine.

**Methods:**

Twenty-six healthy participants and twenty-nine people with migraine participated in the study. PPTs were measured interictally and bilaterally at the cephalic region (temporal muscle, C1 paraspinal muscles, and trapezius muscle) and extra-cephalic region (extensor carpi radialis muscle and tibialis anterior muscle). PPTs were first determined with a digital algometer, followed by an analogue algometer. Intraclass correlation coefficients (ICC_3.1_) and limits of agreement were calculated to quantify concurrent validity.

**Results:**

The concurrent validity between algometers in both groups was moderate to excellent (ICC_3.1_ ranged from 0.82 to 0.99, with 95%CI: 0.65 to 0.99). Although PPTs measured with the analogue algometer were higher at most locations in both groups (*p* < 0.05), the mean differences between both devices were less than 18.3 kPa. The variation in methods, such as a hand-held switch (digital algometer) versus verbal commands (analogue algometer) to indicate when the threshold was reached, may explain these differences in scores. The limits of agreement varied per location and between healthy participants and people with migraine.

**Conclusion:**

The concurrent validity between the digital and analogue algometer is excellent in healthy participants and moderate in people with migraine. Both types of algometer are well-suited for research and clinical practice but are not exchangeable within a study or patient follow-up.

## Background

Pressure pain threshold (PPT) is defined as the amount of pressure applied over the skin when a feeling of pressure transitions into a feeling of painful pressure [1]. PPTs are assessed to determine increased mechanical sensitivity as a feature of central and/or peripheral sensitization in headache research [2, 3]. Significantly lower PPTs are reported in people with migraine compared to healthy participants at the cephalic region [1], but the evidence is inconsistent for the extra-cephalic region [4, 5]. Algometry is not only used in research to elucidate the underlying neurophysiological mechanisms. In clinical practice, PPT measurements are also used to assess mechanical pressure sensitivity in people with headache disorders and evaluate treatment effects [6–9].

Most studies in headache research [1–4] used a digital algometer to measure PPTs. The main difference between a digital and analogue algometer is the use of a digital display versus a dial with a needle to determine the PPT value [10]. Some digital algometers (e.g. Type II, Somedic Electronics, Solna, Sweden) has a hand-held switch which the participant can when the threshold is reached. For some analogue algometers (Force Dial FDK, Wagner Instruments, Greenwich, Connecticut) the examiner responds to a verbal cue from the participant. Furthermore, some digital algometers (e.g., Type II, Somedic Electronics, Solna Sweden) provide visual feedback about the pressure application rate. The pressure can be expressed in kilo Pascals (kPa) or kilogram per square centimetre (kg/cm^2^). Another main difference is the acquisition cost, with an analogue algometer typically being considerably less expensive (e.g., ~$280 for a Wagner analogue algometer and ~ $5.500 for a Somedic digital algometer). The clinimetric properties of analogue and digital algometers that have been reported show excellent construct validity [11], high intrarater [12, 13] and interrater [14, 15] reliability in healthy participants and people with headache [16]. Although both algometers show high construct validity and reliability, we lack information about the concurrent validity between these tools in healthy participants and people with migraine. If researchers and clinicians want to be able to compare the findings in the literature or across clinical practices, they need to know how valid analogue systems are compared to digital systems.

Therefore, this study aimed to estimate the concurrent validity between digital and analogue algometers in healthy participants and people with migraine in cephalic and extra-cephalic regions.

## Methods

### Design

This study was a prospective observational study on healthy participants and people with migraine. The Medical Ethical Committee of the Amsterdam University Medical Centre approved the study (METC-2015-551). Written informed consent was obtained from all participants prior to the start of the measurements.

### Participants

Healthy participants were recruited from the general population via advertising. Healthy participants were excluded if they reported any history of neck pain, headache, chronic pain, psychiatric conditions or neurological disorders in the last 12 months.

In the same period, people with migraine were recruited from primary healthcare clinics in The Netherlands. People with migraine were eligible for inclusion if they were: diagnosed with migraine according to the International Classification of Headache Disorders (ICHD - III) [17], between 18 and 65 years old, and Dutch or English speaking. Exclusion criteria were: participants with other types of headaches, such as medication overuse headache, a non-specific cause of headache, traumatic head/neck symptoms within 2 months before the measurements, chronic musculoskeletal painful conditions, psychiatric conditions, malignancy or neuropathic pain states other than migraine. Participants were not allowed to use analgesic medications or non-steroid anti-inflammatory drugs (NSAID) within 24 h before the measurements or receive treatment within 48 h before the measurements.

### Assessor

One assessor (TD) performed all measurements. During a 2 weeks period, the assessor underwent training for ~ 15 h to learn how to calibrate the instruments, maintain the required rate of pressure increase and apply the standardized protocol. The training PPT measurements were performed on healthy adult volunteers who did not participate in the actual experiment.

### Questionnaires

At baseline, all participants completed a general questionnaire to document sociodemographic information. They scored their current headache intensity on a 10-point Numeric Pain Rating Scale (NPRS, 0 = no pain and 10 = worst possible pain) and completed the Headache Impact Test-6 (HIT-6) questionnaire. The NPRS has been shown to be a valid and reliable instrument in several populations [18]. The HIT-6 questionnaire was used to measure the influence of headache on daily functioning. Scores on the HIT-6 questionnaire range from 36 to 78: higher scores indicate a higher impact of migraine. The HIT-6 questionnaire is a reliable (ICC = 0.77) and valid tool to determine headache impact in a migraine population [19–21].

### Concurrent validity

Healthy participants and people with migraine were measured to determine the concurrent validity at both the cephalic and extra-cephalic regions. Since pain sensitivity differs between sides in people with migraine [21], concurrent validity was determined separately for the dominant and non-dominant side of the migraine. The dominant side was defined as the most painful side of the last migraine attack. The side of the dominant hand was used to define the corresponding ‘dominant side’ in healthy participants. People with migraine were measured during their interictal phase (the phase without headache or other symptoms between two consecutive migraine attacks). To ensure that the examination was in the interictal phase, we contacted the participants before the initial measurement. All participants with migraine were asked when they had their last ictal and post-ictal phase and if they were in a headache-free period for more than 48 h.

### Digital algometer

**Fig. 1:**
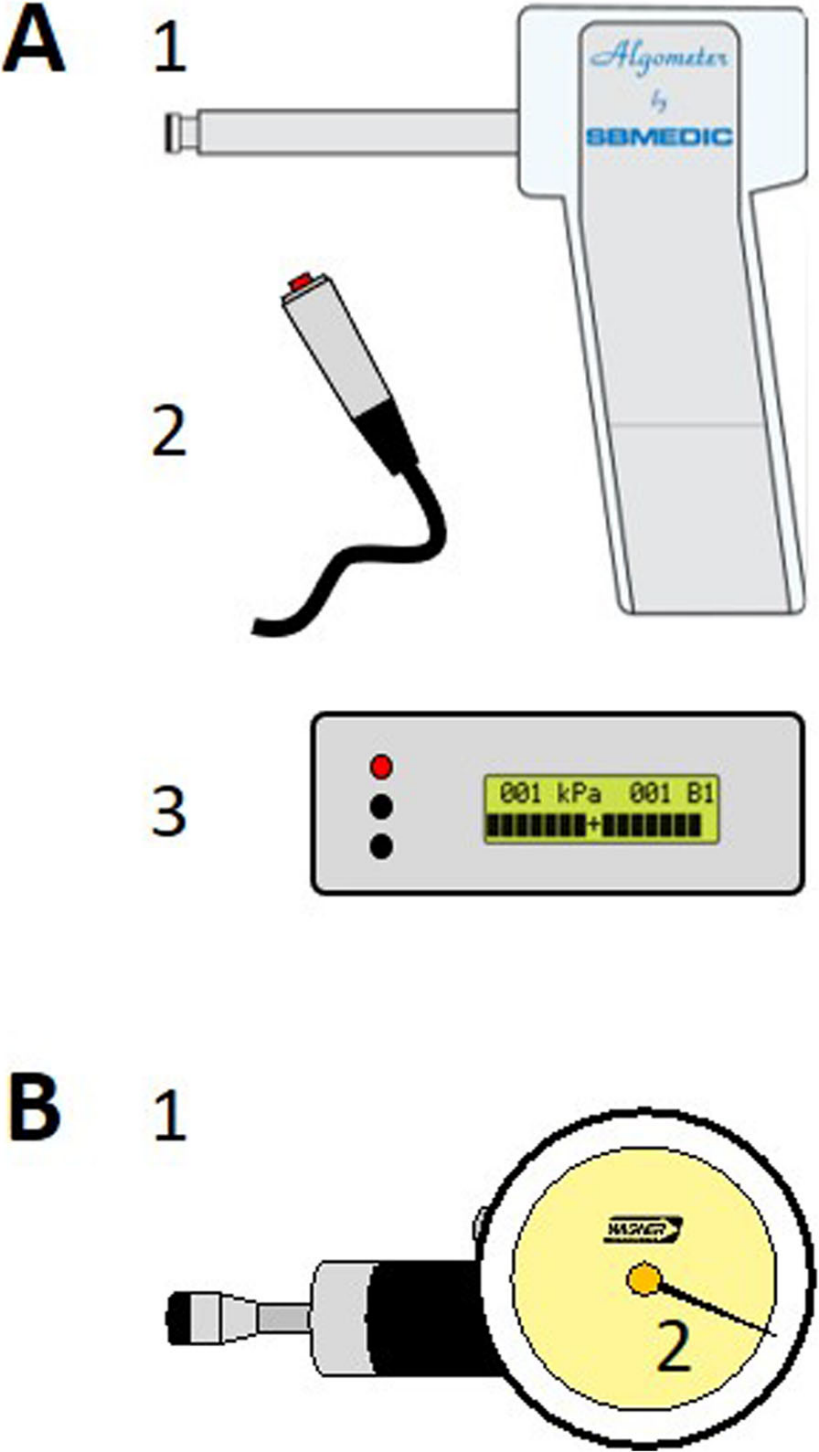
**A** Differences Pressure Pain Thresholds digital and analogue algometer Temporalis muscle dominant side in healthy participants. **B** Differences Pressure Pain Thresholds digital and analogue algometer Temporalis muscle dominant side in people with migraine

A digital algometer (Type II, Somedic Electronics, Solna, Sweden) with a 1 cm^2^ probe was used to measure PPTs (Fig. 1A). The Somedic algometer has excellent construct validity (ICC > 0.98) compared to a force plate [11]. The ICC values of intrarater reliability are ranging from 0.90 to 0.95 in healthy participants and from 0.89 to 0.96 in people with migraine [22, 23].

### Analogue algometer

A Wagner algometer (Force Dial FDK 20, Wagner Instruments, Greenwich. Connecticut) with a 1 cm^2^ probe area was used as an analogue algometer (Fig. 1B). The hand-held Wagner algometer with a digital reading function shows comparable correlations to a force plate (Pearson’s *r* = 0.99) [24]. Previous studies with comparable hand-held analogue algometers showed ICC values for intrarater reliability ranging from 0.79 to 0.91 in healthy participants [14] and from 0.90 to 0.97 for people with chronic tension-type headache [16].

### Measurement procedures

Prior to commencing the experiment, the Somedic and Wagner algometers were calibrated according to the manufacturer’s instructions. To familiarise participants with the procedures, two PPT measurements were performed for both the digital and analogue algometer at the midpoint of the biceps brachii muscle. Throughout the experiment, participant sat in a relaxed position on a chair, in an upright position, with both arms supported by armrest. At the start of the measurement, all locations were marked on the participants’ skin with a black skin marker. Each location was measured three times with the analogue and the digital algometer. There was a 30 min interval between the measurements with the digital and analogue algometer, and a 30s interval between the three consecutive repetitions to minimize wind-up. To reduce variability, we placed the tip of both algometers in the same place, i.e., the footprint of the tip of the algometer at the marked location which remained visible between the 30s repetitions.

PPTs were assessed bilaterally at five test locations in a standardized order: from the most cranial location to the most caudal location, i.e.: (1) temporal muscle (1 cm lateral to the external angle of the orbit), (2) C1 paraspinal muscles (2 cm lateral from the midline of the neck), (3) upper trapezius muscle (on the midpoint between the acromion and spinous process C7), (4) extensor carpi radialis muscle (at 1/3 length of the forearm distal to the elbow) and (5) tibialis anterior muscle (at 1/3 of the length of the lower leg, distal to the knee). In people with migraine, all measures were first performed on the dominant side of the migraine.

PPTs were first measured with the Somedic algometer. The assessor placed the tip of the algometer perpendicular on the skin over the target location and increased the pressure with a rate of 50 kPa/s [3, 25]. Participants were instructed to press the switch when the feeling of pressure changed into the feeling of pain. Once the button was pressed, the measurement stopped automatically. Once all measurements from all locations were obtained with the digital algometer, the measurements with the analogue algometer were performed by the same assessor. Measurements with the analogue algometer were performed at the same locations, visible by the ‘pressure mark’ from the tip of the algometer from the previous measurements. The assessor was trained to increase the pressure with the Wagner algometer at a rate of 0.5 kg/cm^2^/s [3, 25]. Participants had to say ‘stop’ when the threshold was reached as hand-held switches are not available for analogue algometers. After each measurement, the assessor read the pressure from the display of the analogue algometer and entered this score in a datasheet.

### Blinding

The assessor was blinded for the participant’s condition. All participants were blinded for the measurement outcomes. The data analysis was performed by a researcher who was not involved in the measurements.

### Data analysis

The mean of the three PPT measures was calculated for each location for each participant [26]. Values from the analogue algometer were converted into kPa using the following formula: 1 kg-force per square centimetre (kg/cm^2^) = 98.0665 kPa.

### Statistical analysis

Because no sample size calculation for concurrent validity is available [27], we used a comparable number of participants as used in other concurrent validity studies [28, 29]. Statistical analyses were performed using SPSS version 25.0 (SPSS Inc., Chicago, Illinois, USA). Normality of continuous variables was checked by visual inspection of the Q-Q plots and Shapiro-Wilk test. Mean differences between digital and analogue measurement were analyzed with an independent samples t-test. We used a two-way mixed effect, consistency and single rater/measurement model (ICC_3.1_) to quantify the concurrent validity between the algometers [30]. The following cut-off points were used for interpretation of the ICC: < 0.5: poor validity, 0.5–0.75: moderate validity, 0.75–0.9: good validity, and values > 0.90: excellent validity [31]. Additionally, limits of agreement (= *d* ± 1.96 x SD) were calculated with Bland-Altman plots to determine the mean and 95% confidence interval (CI) of the differences between the digital and analogue algometer [32]. The independent samples t-test was performed to test systematic differences in PPTs between the digital and analogue algometer.

## Results

Twenty-six healthy participants (69% female; mean (SD) age: 42.8 (14.3) years) and 29 people with migraine (89% female; 47.8 (10.8) years) were included in this study. Table 1 provides an overview of the participants’ characteristics. There were no missing data.

**Table 1:** Main characteristics at baseline of healthy participants and people with migraine

	Healthy participants (***n*** = 26)	Migraine (***n*** = 29)
**Age (SD)**	42.8 (14.3)	47.8 (10.8)
**Gender (% female)**	18 (69%)	26 (89%)
**Days of migraine per month (SD)**	n/a	6.8 (4.7)
**Episodic migraine %**	n/a	21 (72%)
**Chronic migraine %**	n/a	8 (28%)
**HIT- 6**^**a**^ **(SD)**	42.8 (9.1)	63.3 (7.5)
**NPRS**^**b**^ **headache severity (SD)**	0.3 (0.5)	1.1 (1.4)
**Aura (% yes)**	n/a	6 (20.7)

^a^ HIT 6: Headache Impact Test is ranging from 36 to 78 points; ^b^NPRS: Numeric Pain Rating Scale ranging from 0 to 10 points; n/a: not applicable

Table 2 presents the mean PPT values of the digital and analogue algometer for each test locations at the dominant and non-dominant side of healthy participants and people with migraine. Compared to the digital algometer higher PPT values were obtained with the analogue algometer, indicating a systematic error between the digital and analogue PPT measurements. In both groups, the mean difference in PPT values between both algometers ranged from − 6.3 to 2.3 kPa in healthy participants and from − 18.3 to − 3.5 kPa in people with migraine (Table 3).

**Table 2:** Pressure pain thresholds and concurrent validity in healthy participants and people with migraine

	PPTs in healthy participants (***n*** = 26)			PPTs in people with migraine (***n*** = 29)		
Location	Digital algometer (mean, SD)	Analogue Algometer (mean, SD)	ICC _3.1_ (95%CI)	Digital Algometer (mean, SD)	Analogue Algometer (mean, SD)	ICC _3.1_ (95%CI)
**Dominant side**						
Temporalis muscle	283.3 (57.7)	289.6 (53.2)	.98 (0.96–0.99)	179.1 (45.1)	185.9 (51.6)	.86 (0.73–0.93)
C1 paraspinal muscles	277 (48.7)	280.4 (49.0)	.98 (0.96–0.99)	218.6 (58.2)	227.1 (59.9)	.94 (0.87–0.97)
Trapezius muscle	357.1 (55.5)	361.1 (51.3)	.97 (0.94–0.99)	317.2 (85.6)	332.8 (88.9)	.95 (0.89–0.97)
Extensor carpi radialis muscle	282.0 (52.5)	286.0 (50.9)	.97 (0.94–0.99)	236.4 (56.6)	240.8 (54.3)	.82 (0.65–0.91)
Tibialis anterior muscle	561.7 (77.4)	559.4 (78.5)	.98 (0.96–0.99)	436.9 (121.3)	449.3 (120.6)	.97 (0.95–0.99)
**Non dominant side**						
Temporalis muscle	283.5 (55.8)	289.2 (54.3)	.98 (0.97–0.99)	173.8 (41.2)	177.4 (40.3)	.93 (0.85–0.96)
C1 paraspinal muscles	276.4 (44.2)	282.2 (43.7)	.97 (0.94–0.99)	219.2 (61.3)	230.9 (63.7)	.94 (0.87–0.97)
Trapezius muscle	354.8 (60.0)	361.1 (51.2)	.98 (0.95–0.99)	317.2 (88.9)	335.4 (90.2)	.95 (0.89–0.97)
Extensor carpi radialis muscle	278.2 (50.3)	282.6 (50.9)	.98 (0.95–0.99)	238.4 (58.3)	254.6 (59.3)	.84 (0.70–0.92)
Tibialis anterior muscle	562.7 (75.5)	564.7 (77.5)	.99 (0.98–0.99)	435.1 (115.5)	446.6 (128.7)	.97 (0.94–0.99)

PPT score of digital and analogue algometer (mean, SD) of all locations at the dominant and non-dominant side. Concurrent validity: Intraclass correlation coefficient_3.1_ (95% confidence interval) of digital and analogue algometer

**Table 3:** Mean difference of all locations between the digital and analogue algometer and limits of agreement in people with healthy participants and people with migraine

	Healthy participants (***n*** = 26)				People with migraine (***n*** = 29)			
Location	Mean difference in kPa (SD)	*P* value	Limits of agreement		Mean difference in kPa (SD)	*P* value	Limits of agreement	
			Upper limit	Lower Limit			Upper limit	Lower Limit
**Dominant side**								
Temporalis muscle	−6.3 (10.8)	0.007	14.9	−27.9	− 6.8 (25.3)	0.16	42.8	−56.8
C1 paraspinal muscle	−3.4 (9.6)	0.8	15.4	−21.6	−8.5 (20.8)	0.04	32.3	−49.3
Trapezius muscle	−3.9 (12.4)	0.12	20.4	−28.2	−15.6 (28.6)	0.007	40.5	−71.7
Ext. carpi radialis m.	−4.0 (11.8)	0.09	19.1	−27.1	−4.4 (33.4)	0.49	61.1	−69.9
Tibialis anterior muscle	2.3 (14)	0.4	29.7	−25.1	−12.4 (27)	0.02	40.5	−65.3
**Non dominant side**								
Temporalis muscle	−5.7 (9.4)	0.005	12.7	−24.1	−3.5 (15.6)	0.24	27.1	−34.1
C1 paraspinal muscle	−5.8 (10.6)	0.009	14.9	−26.6	−11.7 (21.9)	0.008	31.2	−54.6
Trapezius muscle	−6.3 (12.2)	0.14	17.6	−30.2	−18.3 (29.6)	0.003	39.7	−76.3
Ext. carpi radialis m.	−4.4 (11)	0.05	17.2	−25.9	−16.2 (32.8)	0.01	48.1	−80.5
Tibialis anterior muscle	−2 (11.2)	0.36	19.9	−23.9	−11.6 (28.6)	0.04	44.5	−67.7

### Concurrent validity

**Fig. 2:**
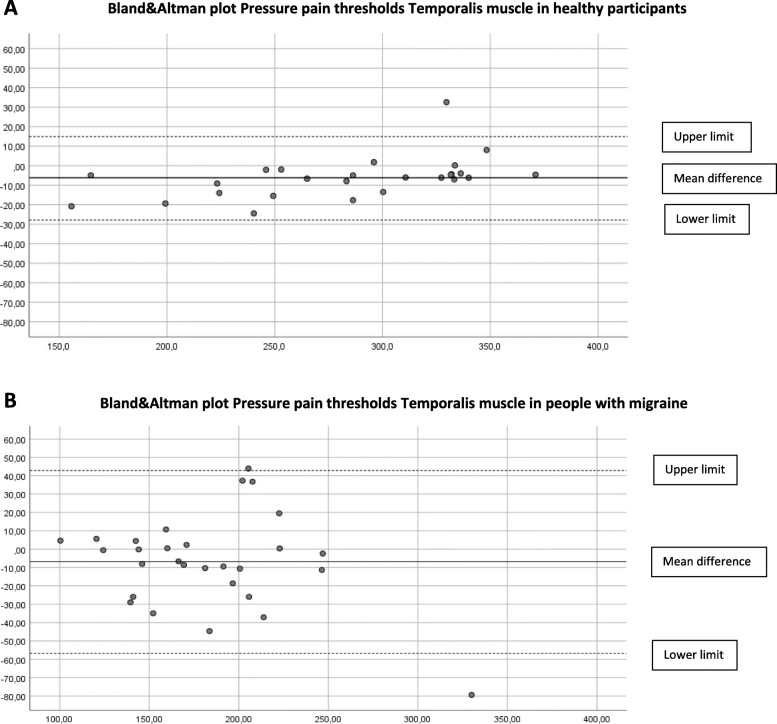
**A** Differences Pressure Pain Thresholds digital and analogue algometer Temporalis muscle dominant side in healthy participants (mean difference, upper and lower limit). **B** Differences Pressure Pain Thresholds digital and analogue algometer Temporalis muscle dominant side in people with migraine (mean difference, upper and lower limit)

**Fig. 3:**
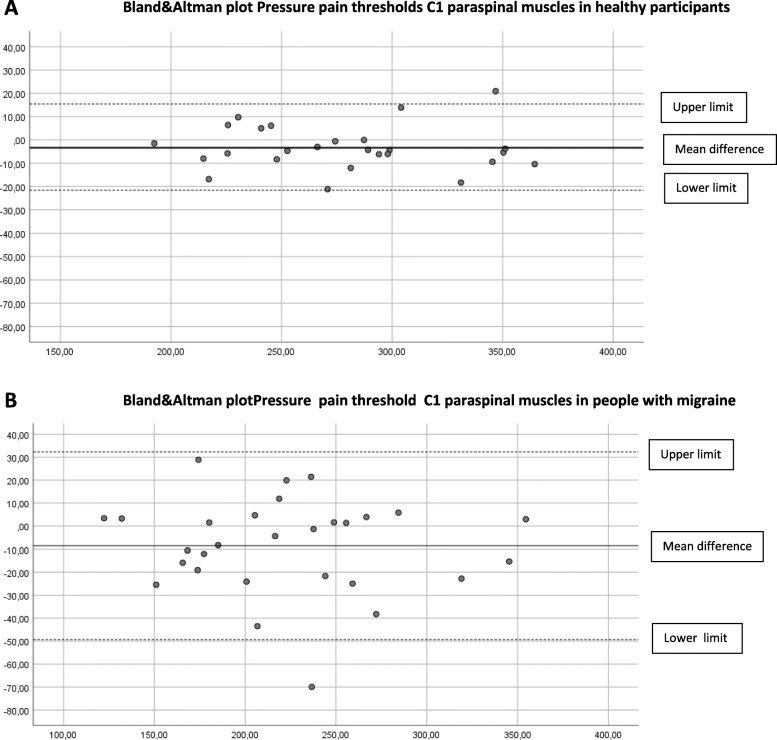
**A** Differences Pressure Pain Thresholds digital and analogue algometer C1 paraspinal muscles dominant side in healthy participants (mean difference, upper and lower limit). **B** Differences Pressure Pain Thresholds digital and analogue algometer C1 paraspinal muscles dominant side in people with migraine (mean difference, upper and lower limit)

**Fig. 4:**
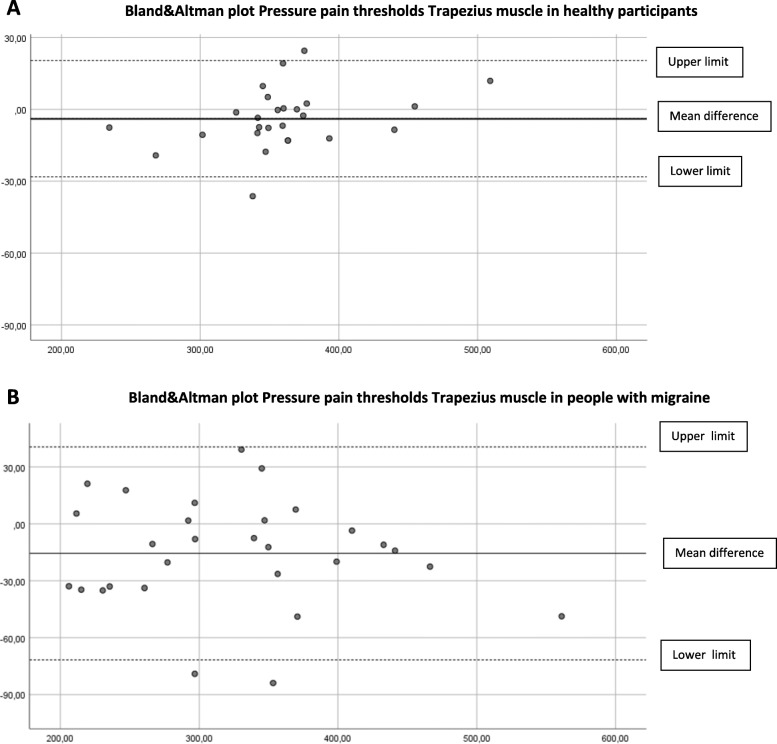
**A** Differences Pressure Pain Thresholds digital and analogue algometer Trapezius muscle dominant side in healthy participants (mean difference, upper and lower limit). **B** Differences Pressure Pain Thresholds digital and analogue algometer Trapezius muscle dominant side in people with migraine (mean difference, upper and lower limit)

**Fig. 5:**
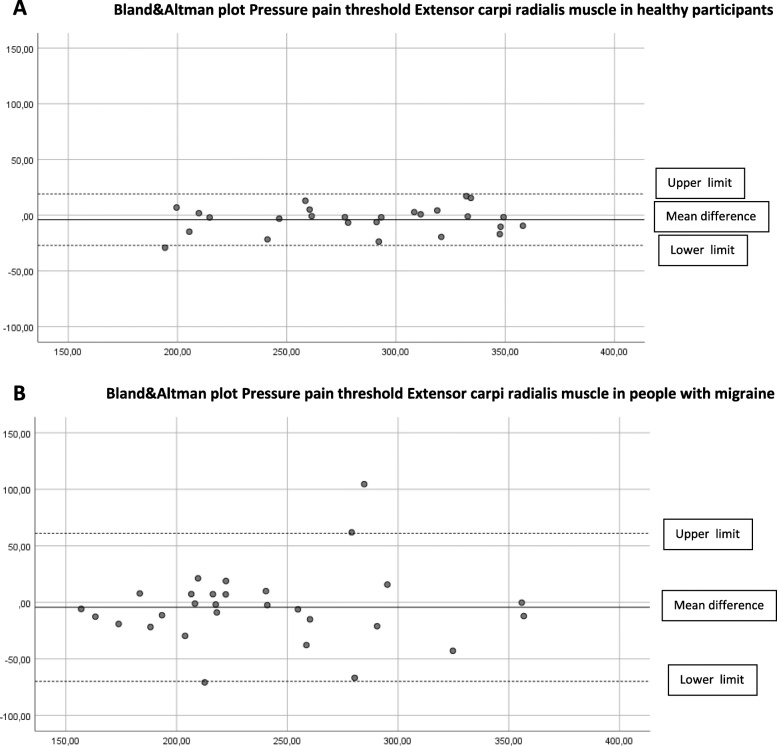
**A** Differences Pressure Pain Thresholds digital and analogue algometer Extensor carpi radialis muscle dominant side in healthy participants (mean difference, upper and lower limit). **B** Differences Pressure Pain Thresholds digital and analogue algometer Extensor carpi radialis muscle dominant side in people with migraine (mean difference, upper and lower limit)

**Fig. 6:**
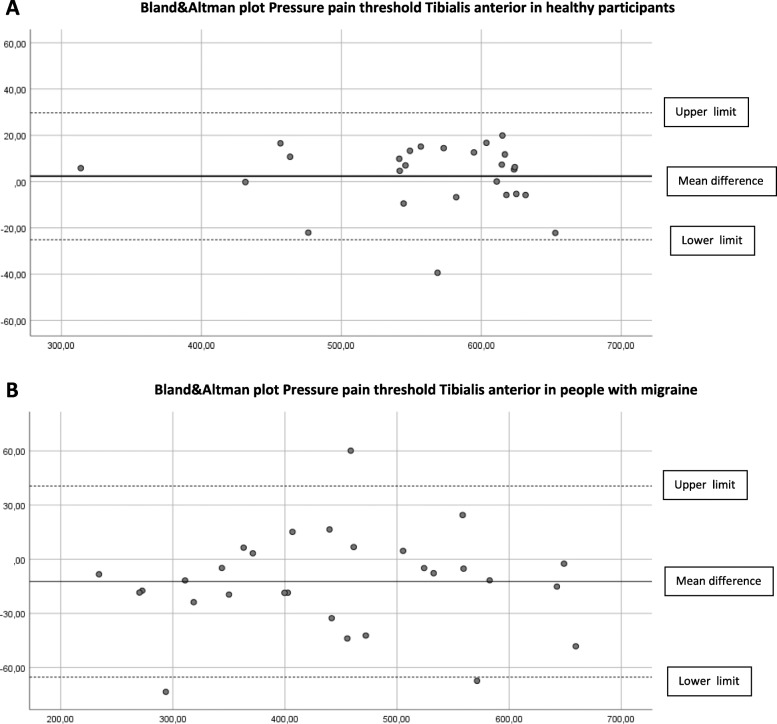
**A**. Differences Pressure Pain Thresholds digital and analogue algometer Tibialis anterior muscle dominant side in healthy participants (mean difference, upper and lower limit) **B**. Differences Pressure Pain Thresholds digital and analogue algometer Tibialis anterior muscle dominant side in people with migraine (mean difference, upper and lower limit)

The concurrent validity in the cephalic and extra-cephalic region in healthy participants was excellent (ICC _3.1_ > 0.97). In people with migraine, the ICCs were good to excellent and ranged from 0.82 to 0.97 (Table 2). Limits of agreement (LoA) are shown in Table 3 and displayed in the Bland & Altman plots (Figs. 2A-B, 3A-B, 4A-B, 5A-B, 6A-B).

## Discussion

The Somedic digital and Wagner analogue algometer showed good to excellent concurrent validity in healthy participants and in people with migraine. At most locations, the PPT scores obtained with the analogue algometer were higher, suggesting a systematic error. Although we found good to excellent concurrent validity, the upper and lower limits of the mean difference between the digital and the analogue algometer reflect a wide range in scores in people with migraine. This means that clinicians have to be aware that PPTs measured with a digital or analogue algometer may differ substantially in people with migraine.

The difference in the method of digital and analogue measurement may have caused the systematic error. When the participant pressed the hand-held switch, the reading of the threshold on the digital algometer stopped immediately. During the test with the analogue algometer, the participant had to give a verbal cue to the assessor. Then, the assessor stopped the test. The time needed for the assessor to stop the test may have resulted in a slightly higher pressure score. This delay in terminating the measurement seems to be the most plausible reason for the systematically higher PPT values retrieved with the analogue algometer. Besides this difference in method of measurement, the fixed order in which we first measured all locations with the digital algometer may have contributed to systematic error. Although randomization of the order of measurement would be preferable, we did not randomize the order of the measurements as this study was part of a larger project evaluating PPTs in people with migraine by using a digital algometer [33]. Randomization of the algometers (analogue versus digital) would have strengthened the design of this study.

Still, randomization of measurement order appears not to be the most important contributing factor to systematic error. In previous research where they applied a random sequence of PPT measurement in the lumbar region in healthy participants, the procedure with a hand-held manual analogue algometry and verbal command also resulted in significantly higher PPT score as compared to computerized algometry using a hand-held stop [34]. Further, both devices show excellent, almost perfect construct validity [11] compared to a force plate. So, the small but significant difference between the digital and analogue PPT values, but also the wide range between the lower and upper limit of agreement between both measurements, seems more likely to depend on method of measurement and the participant’s condition. Especially in people with migraine, we suspected that a wind-up phenomenon would be detectable due to consecutive PPT measurements, resulting in lower analogue PPT scores. Our results did not confirm this assumption. In contrast, we found higher analogue PPT values in both groups. The Bland-Altman plots revealed no proportional bias as with increased pressures, no larger (or smaller) differences were observed between the two devices.

The described variation in mean difference and limits of agreement of location between the groups can be explained by a larger variation in PPT scores in the group of people with migraine. Except for the temporalis muscle, all locations show larger mean differences in mean PPT scores in the migraine group. This difference in mechanosensitivity in people with migraine may explain the variation in mean differences and limits of agreement between the groups.

Only one assessor performed all measurements. This assessor was trained to perform the measurements and was selected from a pool of physiotherapy students enrolled in a master program. Although previous research showed high values for intratester and interrater reliability in novice and experienced assessors [35, 36], the lack of information on reliability of the measurement may hamper the interpretation of our results.

For interpretation of the ICC, we applied widely accepted cut-off points as described by Koo et al. [31]. According to their recommendation, we included the confidence interval around the ICC in the reported cut-off point. The concurrent validity of the digital and analogue algometer was excellent in healthy participants because ICC values ranged from 0.96 to 0.99 with the lower boundaries of the confidence intervals not below 0.90. For people with migraine, both algometers showed moderate to excellent ICC values ranging from 0.82 to 0.97 with the lowest boundary of the confidence interval not below 0.65.

Since the ICC _3.1_ is not sensitive to systematic errors, the Bland-Altman plots and LoA are essential to judge if simultaneous or interchangeable use of both algometers is applicable in clinical practice and research. Based on our study, clinicians must be aware of systematic bias as visualized in the Bland-Altman plots and the wide range between the upper and lower limits of agreement in people with migraine.

To determine a difference in PPT measurement using both algometers, a difference of more than 37–55 kPa in healthy participants and a substantial difference of more than 81–130 kPa in people with migraine has to be detected, depending on the measured test location. Therefore, to minimize measurement error, we conclude that both algometers are not interchangeable to measure PPTs in people with migraine.

We have studied the concurrent validity of a digital (Type II, Somedic Electronics, Solna, Sweden) and analogue algometer (Force Dial FDK, Wagner Instruments, Greenwich, Connecticut). There are other digital algometers, such as the AlgoMed (Medoc) and analogue algometers, available. Because the method of measurement may differ between devices, we have to be careful to generalize our findings to other brands of algometers.

## Conclusion

The digital and analogue algometers show moderate to excellent concurrent validity in healthy participants and people with migraine. We recommend clinicians and researchers to use the same type of algometer in clinical practice and research, for both cross-sectional and longitudinal use. The analogue algometer can be considered as a valid, easy to handle, and low-cost instrument to assess mechanical sensitivity in healthy participants and people with migraine.

## Data Availability

The datasets used and/or analyzed during the current study are available from the corresponding author on reasonable request.

## Abbreviations

ICC: Intraclass correlation coefficient
PPT: Pressure pain threshold
kPa: Kilo Pascal
kg/cm^2^: Kilogram per square centimetre
ICHD – III: International Classification of Headache Disorders III
